# Aurora kinase A in gastrointestinal cancers: time to target

**DOI:** 10.1186/s12943-015-0375-4

**Published:** 2015-05-20

**Authors:** Ahmed Katsha, Abbes Belkhiri, Laura Goff, Wael El-Rifai

**Affiliations:** Department of Surgery, Vanderbilt University Medical Center, 760 PRB, 2220 Pierce Avenue, 37232-6308 Nashville, TN USA; Department of Cancer Biology, Vanderbilt University Medical Center, Nashville, TN USA; Department of Hematology, Department of Oncology, Vanderbilt University Medical Center, Nashville, TN USA; Department of Veterans Affairs, Tennessee Valley Healthcare System, Nashville, TN USA

**Keywords:** Aurora kinases, Therapy, AURKA inhibitors, MNL8237, Alisertib, Gastrointestinal, Cancer, Signaling pathways

## Abstract

Gastrointestinal (GI) cancers are a major cause of cancer-related deaths. During the last two decades, several studies have shown amplification and overexpression of Aurora kinase A (AURKA) in several GI malignancies. These studies demonstrated that AURKA not only plays a role in regulating cell cycle and mitosis, but also regulates a number of key oncogenic signaling pathways. Although AURKA inhibitors have moved to phase III clinical trials in lymphomas, there has been slower progress in GI cancers and solid tumors. Ongoing clinical trials testing AURKA inhibitors as a single agent or in combination with conventional chemotherapies are expected to provide important clinical information for targeting AURKA in GI cancers. It is, therefore, imperative to consider investigations of molecular determinants of response and resistance to this class of inhibitors. This will improve evaluation of the efficacy of these drugs and establish biomarker based strategies for enrollment into clinical trials, which hold the future direction for personalized cancer therapy. In this review, we will discuss the available data on AURKA in GI cancers. We will also summarize the major AURKA inhibitors that have been developed and tested in pre-clinical and clinical settings.

## Introduction

**Figure 1:**
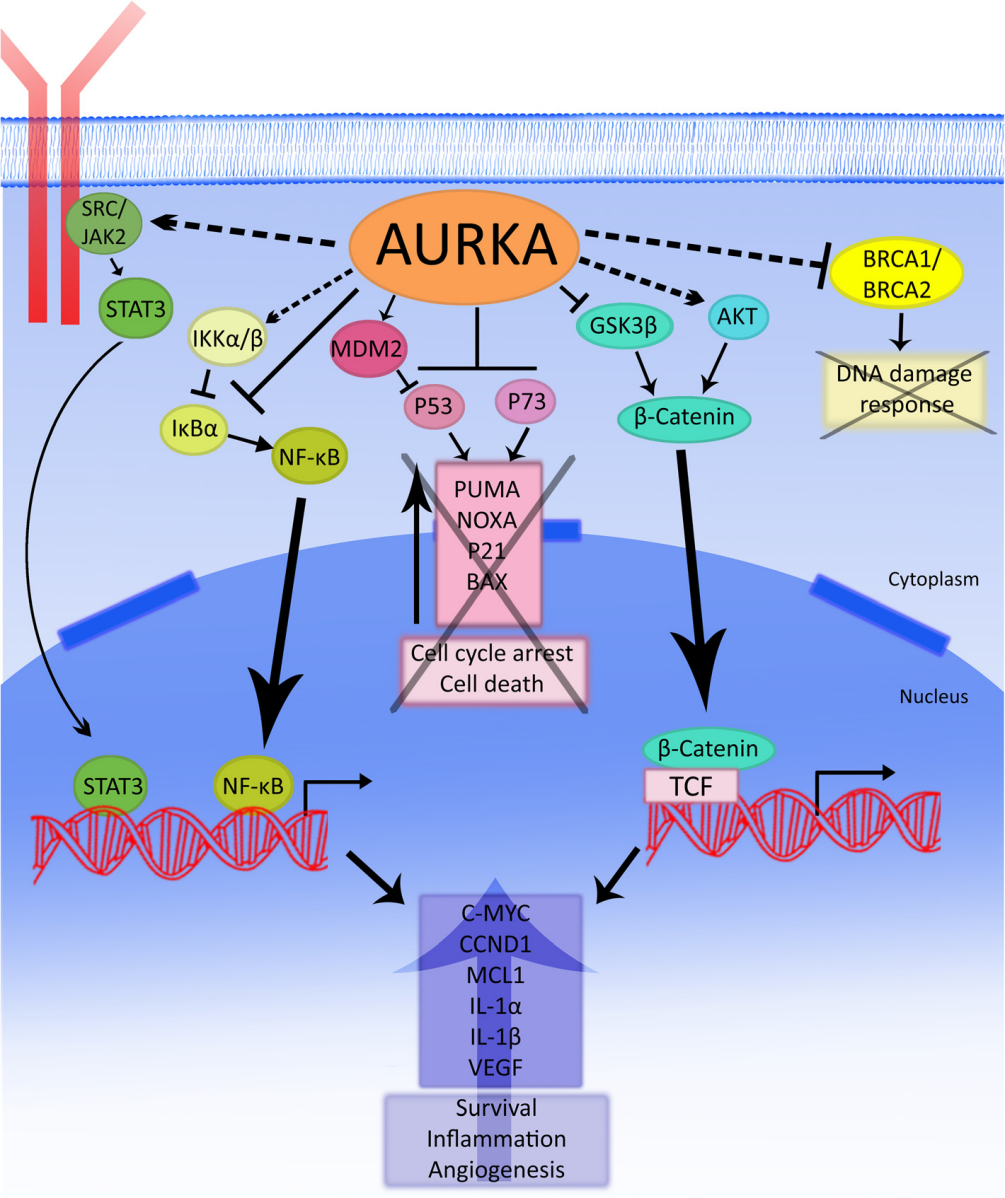
A summary of signaling networks of AURKA in cancer. Solid lines indicate direct regulation, whereas dashed lines indicate indirect regulation.

Mitotic kinases are the main proteins that coordinate accurate mitotic processing [1]. In lower organisms, mitotic kinases include polo-like kinases, NIMA-related kinases, cyclin-dependent kinase 1, WARTS/LATS1-related kinases, and Aurora/Ipl1-related kinases [2]. In humans, the aurora kinase family is considered homologous to Aurora/Ipl1 kinases [2]. This family consists of highly conserved serine-threonine kinases, which play a critical role in regulation of mitotic events like spindle assembly, function of centrosomes and cytoskeleton, and cytokinesis [3,4]. Members of this family are Aurora kinase A (AURKA), Aurora kinase B (AURKB), and Aurora kinase C (AURKC) [3,4]. AURKA is located at chromosome 20q13.2 [5,6] and was found to play an important role during mitosis. It has been shown that AURKA knockdown suppresses centrosome maturation [7,8]. In mice, AURKA genetic ablation or null mutation leads to mitotic arrest and embryonic death at the blastocyst stage [9-11]. Furthermore, AURKA is critical for bipolar-spindle assembly where it interacts with Ran-TPX2 pathway [12]. In addition to its role in mitosis, AURKA is recently reported to regulate stem cell self-renewal, reprogramming, and differentiation [13,14]. Overexpression of AURKA has been reported to impair DNA damage repair through inhibition recruitment of RAD51 to double strands breaks [15]. Additionally, AURKA was found to suppress BRCA1 and BRCA2, both play a major role in DNA damage response [16,17]. Furthermore, AURKA was also reported to regulate cell migration and adhesion [18] and to mediate an increase in VEGF mRNA expression [19]. AURKA activity is dependent on its phosphorylation at the 288-threonine residue (T288); located on the activation loop of the kinase. AURKA is activated through an autophosphorylation process upon binding with TPX2 [12,20], along with several other kinases. AURKA signaling networks are summarized in Table 1 and Figure 1.

**Table 1:** **Selected cancer associated proteins regulated by AURKA**

**Protein**	**Type of regulation**	**Functional significance**	**References**
p53	Phospho-Ser215	Abrogates transactivation	[48,56]
	Phospho-Ser315	Abrogates transactivation and enhances proteasomal degradation	
NF-κBp65	Phospho-Ser536	Promotes inflammation and cell survival	[49]
P73	Phospho-Ser235	Induces apoptosis	[52-56]
MDM2	Phospho-Ser166	Activates MDM2 and degradation of P53	[48]
GSK3β	Phospho-Ser9	Inhibits GSK3β and enhances β-catenin activity	[19]
IκBα	Phospho-Ser32	Induces degradation of IκBα and activation of NF-κB pathway	[49]
JAK2	Increased expression	Activates STAT3	[55]
N-MYC	Increased expression	Stabilizes N-MYC protein	[106]
SRC	Phospho-Tyr416	Activates SRC and promotes cancer cell invasion	[18]
IKKα/β	Phospho-Ser176/180	Activates NF-κB pathway	[107]
NF-κB2/p100	Phospho-Ser866/870	Activates non-canonical NF-κB pathway	[108]
STAT3	Phospho-Tyr705	Promotes cell survival	[55]
RalA	Phospho-Ser194	Enhances transformed cell growth	[74]
BRCA1/BRCA2	Decreased expression	Down-regulate cell cycle and DNA damage response	[16,17,109]

Several studies have shown that AURKA is frequently amplified in several tumors including breast, leukemia, bladder, ovarian, gastric, esophageal, liver, colorectal, and pancreatic cancers [21-30]. Notably, AURKA overexpression has been significantly associated with high-grade (grade II-IV) and high-stage (stage IIIB-IV) hepatocellular carcinoma tumors [30]. Furthermore, it was suggested that AURKA is an essential mediator of chemoresistance in colorectal and breast cancers [31,32]. Importantly, several studies indicated that AURKA overexpression directly leads to malignant transformation and tumor formation [25,33,34]. Additionally, AURKA inhibition or knockdown was shown to induce senescence in multiple myeloma [35] and colon cancer cells [36]. Together, these findings strongly suggest the key role of AURKA in tumorigenesis, and thus, underscore AURKA as an attractive target for cancer therapy.

Gastrointestinal (GI) Cancers are a leading cause of cancer-related deaths in the world. In fact, gastric cancer is the second cause of cancer deaths [37], whereas esophageal neoplasm is the eighth most common cancer worldwide and its incidence, especially adenocarcinoma type, is continuously increasing [38]. At the same time, colorectal cancer is the third most common cancer worldwide [39]. In 2013, more than 30,000 new cases of liver cancer were estimated in men and women in the United States [40]. Similarly, more than 45,000 new cases of pancreatic cancer were estimated in the US in 2014 while its survival rate hardly improved in the last three to four decades [40]. The development of new treatment modalities and targeted therapy approaches, based on the molecular features of the tumor, is urgently needed to improve the dismal survival rates of these highly prevalent cancers worldwide. This review will focus on AURKA as a potential molecular therapeutic target in GI cancers. It will also summarize the recent advancement of AURKA inhibitors in pre-clinical studies and clinical trials.

### AURKA in gastrointestinal cancers

Cancers can develop in any anatomical site of the GI system including esophagus, stomach, small intestine, colon, rectum, liver, gall bladder, and pancreas. A cancer in any of these organs represents a unique type where different molecular mechanisms of tumorigenesis can exist. Recent studies have shown that AURKA is frequently overexpressed and/or amplified in several GI cancers [25-28]. In the following sections, we will review and discuss some of the recently reported studies about AURKA functions in GI cancers.

### AURKA in upper gastrointestinal cancers

Upper gastrointestinal (UGI) cancers include tumors of the stomach and esophagus. While esophageal squamous cell carcinoma (ESCC) is the predominant histological type of esophageal cancers in the world, there has been a significant increase in the incidence of gastroesophageal reflux-related esophageal adenocarcinomas (EAC) in the Western world [41]. The majority of gastric cancers are adenocarcinomas; other subtypes such as lymphomas and gastrointestinal stromal tumors are rare and will not be discussed in this review. Amplifications of chromosomal region 20q13, where AURKA is located, have been described in gastric and esophageal cancers [42,43]. Polymorphisms in the AURKA gene have also been reported in UGI cancers [44,45]. Several studies have shown frequent overexpression of AURKA at the mRNA and protein levels in ESCC, EAC, and gastric cancers [28,46-49]. It has also been suggested that the increased AURKA expression might be used as a prognostic marker of ESCC [50]. In addition, overexpression of AURKA in premalignant and malignant histological stages of reflux-induced Barrett’s tumorigenesis has been reported [51]. Similarly, overexpression of AURKA has been observed in early pre-neoplastic stages of gastric cancer in mouse models and human [49]. Taken together, these reports strongly suggest a critical role for AURKA in carcinogenesis.

Molecular studies have shown that AURKA promotes cell survival signaling while it suppresses the pro-apoptotic machinery in UGI cancer cells [52,53]. We and others have reported that AURKA promotes activation of the AKT pro-survival signaling pathway [47,54]. Furthermore, it has been shown that AURKA can regulate and suppress GSK3β kinase activity in gastric cancer cell lines [19]. The AURKA-GSK3β interaction activates β-catenin/TCF transcription complex, which leads to increased mRNA expression of several oncogenic proteins such as CCND1, c-MYC, c-MYC-binding protein, CLDN1, FGF18, and VEGF [19]. Pro-inflammatory signaling is a significant component in the etiology of tumor development and progression of UGI cancers. In this context, recent reports indicated that AURKA overexpression promotes activation of NF-κB [49] and STAT3 [55] pathways in these cancers. In these studies, AURKA inhibition abrogates the pro-survival and pro-inflammatory signaling mediated by NF-κB and STAT3 with suppression of key pro-survival target genes such as *BCL-xL*, *BCL2*, and *MCL1* [49,55]. Some of the main features of cancer cells include resistance to pro-apoptotic stimuli and the ability to overcome genotoxic stress and chemotherapeutic-induced death. We and others have shown that AURKA can suppress p53 pro-apoptotic functions [48,56]. A recent study has shown that AURKA regulates the key ubiquitin ligase involved in degradation of p53, HDM2, whereby overexpression of AURKA enhanced HDM2 stabilization and promoted degradation of p53, thereby abrogating p53 pro-apoptotic function in response to chemotherapy [48]. Not only does AURKA circumvent p53 pathway, but it also provides a mechanism for cancer cells to evade apoptosis by suppressing p73, an important p53 family protein [52,56]. Collectively, these signaling mechanisms mediated by AURKA can promote cancer cell survival and provide a chemoresistance phenotype. These findings also suggest AURKA as part of a signaling hub controlling several key pathways that regulate the hallmarks of the cancer cell network.

Several AURKA inhibitors have been developed to overcome AURKA-mediated pro-survival and anti-apoptotic activities in cancer cells. Inhibition of AURKA using investigational MLN8054 or MLN8237 led to reversal of anti-apoptotic signaling cascades with activation of pro-apoptotic p73 in p53 deficient cells with re-expression of several pro-apoptotic proteins such as PUMA, NOXA, and p21 [52]. AURKA inhibition by small molecule MLN8237 as a single agent or in combination with Cisplatin or Docetaxel significantly enhanced cell death in esophageal adenocarcinoma xenograft mouse model [53,57]. These findings strongly indicate that AURKA could be an important therapeutic target in upper GI cancers.

### AURKA in colorectal cancer

Colorectal cancer (CRC) was one of the first cancers found to have AURKA amplification. A 1998 study showed that overexpression of *AURKA* resulted in centrosome amplification, chromosomal instability and transformation in mammalian cells, including colon cancer cells [34]. Genetic variations in the *AURKA* gene were also detected and associated with aneuploidy in human colon tumors and low penetrance CRC susceptibility factor [58,59]. AURKA overexpression was associated with the down-regulation of checkpoint with forkhead and ring finger domains (CHFR) in colorectal cancers with high microsatellite instability [60]. Importantly, recent reports indicated that AURKA is critical for tumorigenicity and chemoresistance in CRC stem cells [31], and suggested AURKA as a predictive marker for recurrence in stage III in colon tumors lacking microsatellite instability [61]. These studies, as well as other studies, focused mainly on the fact that overexpression of AURKA leads to chromosomal instability. In normal cells, expression of AURKA protein is predominantly confined to the nucleus and subject to spatio-temporal regulation during mitotic progression. In contrast, expression of AURKA protein is frequently observed in the cytoplasm, in addition to the nucleus, in cancer cells, suggesting an extended role of AURKA beyond regulation of mitosis [62]. This finding provides additional evidence supporting the wealth of signaling data for AURKA reported in upper GI cancers (summarized in the section above). For instance, Tseng and colleagues found that AURKA enhances formation and aggregation of mutant Ras (RasV12) through regulation of RAS/MEK/ERK pathways and AKT phosphorylation in colon cancer [63]. Aurora kinase pharmacological inhibition sensitized colon cancer cells to Tumor necrosis factor (TNF) and TNF-related apoptosis-inducing ligand (TRAIL) [64]. In addition, AURKA inhibition, using specific or pan Aurora kinase inhibitors, sensitized colon cancer cells to chemotherapy [65], TNF or TRAIL induced apoptosis [64] as well as mitotic failure and cell death when inhibited along with SRC [66]. Other reports showed that pharmacological inhibition of AURKA with MLN8054 in colon cancer HCT-116 xenografts induces senescence *in vivo* [36]. These findings suggest that more studies are needed to better characterize the role of AURKA in colon cancer signaling and therapeutics.

### AURKA in pancreatic cancer

Overexpression of AURKA was reported in pancreatic cancer [26], in the early stages of abnormalities in pancreatic ducts and ductal dysplasia in transgenic mouse model for pancreatic cancer [67], and linked to chromosome instability and centrosome abnormality [68]. As observed in other GI cancers, suppression of AURKA using RNA interference [69] or small molecule inhibitor SNS-314 [70] suppresses tumor growth and enhances chemosensitivity. Similar results were obtained with specific AURKA inhibitor CYC3 in combination with Paclitaxel [71]. In addition, AURKA plays a role in protecting human pancreatic ductal adenocarcinoma cells from autophagic cell death induced by metabolic stress [72]. Interestingly, while it was shown in colorectal cancer that AURKA regulates MEK/ERK pathway [63], it was reported that AURKA is a downstream target of this pathway in pancreatic cancer [73], raising the possibility of an existing positive feedback loop between AURKA and MEK/ERK pathway. This signaling loop further underscores the potential therapeutic benefit of targeting AURKA in pancreatic cancer. Additionally, AURKA can phosphorylate RalA at Ser194 and regulate its subcellular localization, which is essential for Ras-mediated cellular transformation [74]. However, further studies by the same group failed to show correlation of RalA (S194) phosphorylation with responsiveness to MLN8237, indicating that the principal target of AURKA may not be RalA [75].

### AURKA in liver cancer

In human hepatocellular carcinoma (HCC), AURKA was found to be overexpressed at mRNA level in 61% of HCC samples [30], whereas gene amplification was recorded in only 3 out of 224 HCC samples [30]. Notably, high expression levels of AURKA were associated with high-grade, high-stage tumors and poor outcome. Microarray studies suggested that AURKA phosphorylates and stabilizes hepatoma up-regulated protein (HURP) [76], a cell cycle regulated gene that is overexpressed in human hepatocellular carcinoma (HCC) [77]. On the other hand, it was reported that hypoxia and hypoxia-inducible factor-1 (HIF-1) are important in the tumor microenvironment and could regulate AURKA at the transcriptional level [78,79]. Other studies showed that AURKA, can in turn, regulate HIF-1α through activation of AKT and p38-MAPK signaling pathways leading to malignant phenotypes of HCC [80], indicating a positive feedback loop sustaining continuous activation of AURKA. Several preclinical studies investigated the effects of AURKA inhibition in HCC. For example, a report showed that the pan Aurora inhibitor PHA-739358 inhibits HCC tumor growth in a tumor xenograft mouse model [81]. Further, AURKA inhibition with small molecule R1498 was found to target mitosis pathway and suppress angiogenesis [82]. These studies emphasize the multifaceted roles of AURKA in HCC and imply AURKA as a feasible druggable target in HCC.

### Clinical development of aurora kinase A inhibitors

The aforementioned preclinical studies shed light on the roles and contributions of AURKA in tumorigenesis, which serve as premise for the development of several pan Aurora kinase inhibitors (AKIs) and selective inhibitors of AURKA. In the following section, we will focus on the present investigational AURKA inhibitors in completed, ongoing, or planned clinical trials.

### MLN8237 (alisertib)

MLN8237 (alisertib) is a second generation derivative of the initial small molecule MLN8054, developed by Millennium Pharmaceuticals, Inc. Both generations work as highly specific ATP-competitive AURKA inhibitors with an IC50 of 2 nM in chronic myeloid leukemia (CML) for MLN8237 [83,84]. The MLN8237 IC50 values vary in other cancers, ranging from 6.7 nM in HeLa cells to 469 nM in DLD-1 colon cancer cell line [85]. MLN8237 can target AURKB at higher doses (396.5 nM) [35,85]. The first generation MLN8054 was terminated in phase 1 clinical trials due to off-target toxicities [86]. Both drugs are ATP competitive, where they bind and inhibit AURKA phosphorylation at T288. In murine xenograft models, MLN8237 was well tolerated with doses ranging from 15 to 40 mg/kg/d. MLN8237 was also found to be well absorbed and steady state plasma levels were reached within 7 days of twice daily dosing in phase 1 clinical trials [87]. Investigations of MLN8237 in preclinical studies have shown that it induces abnormal G2/M cell cycle arrest, abnormalities in mitotic spindles, and apoptosis in upper GI cancers [48,49,53]. In addition, MLN8237 down-regulates several oncogenic pathways such as NF-κB [49,88], AKT [19], and STAT3 [55] in cancer cells. On the other hand, several reports demonstrated that treatment with MLN8237 could activate p53 [47,48,89] and p73 [52,56] apoptotic pathways. The effects of MLN8237 were tested in several non-GI cancers. It has been shown that MLN8237 sensitized PC3 and DU145 prostate cancer cells to radiation, increased the DNA double-strand breaks, induced G2/M cell cycle arrest and polyploidy [90]. Treatment of OVCAR-5 and SKOV3ip2 ovarian cancer cells with MLN8237 resulted in inhibition of cytoskeletal regulatory SRC (assessed by decreased phosphorylation at Tyr-416) and decreased migration and adhesion [18]. Moreover, MLN8237 has been tested on many pediatric cancer cell lines like Ewing sarcoma, neuroblastoma, glioblastoma, rhabdomyosarcoma, ALL, and AML where it inhibited cell growth and delayed tumor growth [91]. *In vitro* and *in vivo* tumor xenografts mouse models showed that MLN8237 enhances anti-tumor activity of chemotherapeutics. For instance, dual treatment of MLN8237 with Docetaxel enhanced apoptosis and anti-tumor activity in lymphoma [92] and UGI adenocarcinomas cell models [53]. Similarly, a combination of Cisplatin with MLN8237 had robust anti-tumor activity in tumor xenograft models of esophageal adenocarcinomas [57]. In B-cell Non-Hodgkin Lymphoma, MLN8237 in combination with Vincristine and Rituximab showed robust cell death *in vitro* and had curative effects *in vivo* using mantle cell lymphoma (MCL) mouse xenograft model [93]. Collectively, the present data indicate that MLN8237 is an effective drug with high specificity and limited off target activity that works in various models. Notably, there are 49 clinical trials with MLN8237 in leukemia, breast, lung, colorectal, ovarian, and pancreatic cancers (clinicaltrials.gov; March 2015). Phase 1 clinical studies of the drug as a single agent demonstrate preliminary activity where 37% of patients achieved a best response of stable disease for more than 6 months in some solid tumors (colorectal cancer, chondrosarcoma, leiomyosarcoma, and liposarcoma) with modest toxicity such as nausea, stomatitis, and alopecia among other adverse effects [87]. The recommended phase 2 dose of MLN8237 is 50 mg orally given twice daily on days 1–7 of a 21-day cycle for various solid tumors and hematologic malignancies [94]. Phase 2 studies of MLN8237 as a single agent are ongoing in multiple tumor types including lung, breast, ovarian, UGI, and pancreatic cancers (Table 2). In some of the clinical trials, MLN8237 has been tested in combination with other drugs such as Cetuximab and Docetaxel among others (Table 2). A recent phase 2 clinical trial of MLN8237 as a single agent in advanced solid tumors revealed that 9% of patients (4 out of 47 patients) with gastro-esophageal adenocarcinoma partially responded to the treatment [95]. A newly registered phase 1 clinical trial (NCT02319018) is ongoing to investigate the side effects of MLN8237 in combination with other chemotherapeutic drugs (fluorouracil, leucovorin calcium, oxaliplatin). This clinical trial will be the first to evaluate MLN8237 exclusively in gastrointestinal tumors (clinicaltrials.gov, Table 2).

**Table 2:** **Selected clinical trials of Aurora kinases inhibitors**

**Inhibitor**	**Chemical structure**	**Target, selectivity (IC50)**	**NCT number, phase, status**	**Cancer type**	**Ref.**
MLN8237		AURKA (2 nM)	NCT01898078, Phase 1, Ongoing	Advanced Solid Tumors; Lymphoma	
			NCT01045421, Phase 1/Phase 2, Completed	Nonhematological Malignancies	[95]
			NCT01091428 Phase 1/Phase 2, Ongoing **(Agent in combination with Paclitaxel)**	Ovarian Carcinoma; Fallopian Tube Cancer; Peritoneal Cancer; Breast Carcinoma	
			NCT01471964, Phase 1/Phase 2, Ongoing **(Agent in combination with Erlotinib)**	Metastatic or Recurrent Non-Small Cell Lung Cancer	
			NCT02114229, Phase 2, Ongoing	Malignant Rhabdoid Tumor; Atypical Teratoid Rhabdoid Tumor	
			NCT00962091, Phase 1, Completed	Advanced Solid Tumors	
			NCT01512758, Phase 1, Completed	Adult East Asian Patients With Advanced Solid Tumors or Lymphomas	
			NCT02109328, Phase 2, Ongoing	Bladder Cancer; Transitional Cell Carcinoma	
			NCT01812005, Phase 2, Ongoing **(Agent in combination with Rituximab)**	Relapsed or Refractory B-Cell Non-Hodgkin Lymphoma	
			NCT01397825, Phase 1/Phase 2, Ongoing **(Agent in combination with Rituximab and Vincristine)**	Relapsed or Refractory Aggressive B-Cell Lymphoma	
			NCT00500903, Phase 1, Completed	Advanced Solid Tumors	
			NCT00697346, Phase 1, Completed	Advanced Hematological Malignancies	[110]
			NCT01799278, Phase 2, Ongoing	Metastatic Castrate Resistant and Neuroendocrine Prostate Cancer	
			NCT00807495, Phase 2, Completed	Aggressive Non-Hodgkin’s Lymphoma	
			NCT01567709, Phase 1, Ongoing **(Agent in combination with Vorinostat)**	Relapsed or Recurrent Hodgkin Lymphoma, B-Cell Non-Hodgkin Lymphoma, or Peripheral T-Cell Lymphoma	
			NCT00651664, Phase 1, Completed.	Advanced Solid Tumors; Lymphomas	
			NCT01695941, Phase 1, Ongoing **(Agent in combination with Bortezomib and Rituximab)**	Relapsed or Refractory Mantle Cell Lymphoma or B-Cell Low Grade Non-Hodgkin Lymphoma	
			NCT01154816, Phase 2, Completed	Recurrent or Refractory Solid Tumors or Leukemia	
			NCT02038647, Phase 2, Ongoing **(Agent in combination with Placebo and Paclitaxel)**	Small Cell Lung Cancer	
			NCT01779843, Phase 1, Ongoing **(Agent in combination with Cytarabine and Idarubicin)**	Acute Myeloid Leukemia	
			NCT00830518, Phase 2, Completed	Acute Myelogenous Leukemia and High-Grade Myelodysplastic Syndrome	
			NCT01677559, Phase 1, Ongoing **(Agent in combination with Nab-Paclitaxel)**	Advanced Solid Malignancies	
			NCT01924260, Phase 1, Ongoing **(Agent in combination with Gemcitabine)**	Solid Tumors or Pancreatic Cancer	
			NCT01897012, Phase 1, Ongoing **(Agent in combination with Romidepsin)**	Relapsed or Refractory B-Cell or T-Cell Lymphomas	
			NCT01034553, Phase 1/Phase 2, Ongoing **(Agent in combination with Bortezomib)**	Relapsed or Refractory Multiple Myeloma	
			NCT01482962, Phase 3, Ongoing **(Agent in combination with Pralatrexate, Gemcitabine and Romidepsin)**	Relapsed/Refractory Peripheral T-Cell Lymphoma	
			NCT01848067, Phase 1/Phase 2, Ongoing **(Agent in combination with Abiraterone acetate and Prednisone)**	Hormone-Resistant Prostate Cancer	
			NCT01466881, Phase 2, Ongoing	Relapsed or Refractory Peripheral T-Cell Non-Hodgkin Lymphoma	
			NCT01923337, Phase 1, Ongoing **(Agent in combination with Irinotecan hydrochloride)**	Advanced Solid Tumors or Colorectal Cancer	
			NCT01714947, Phase 1, Completed	Advanced Solid Tumors; Lymphoma	
			NCT01094288, Phase 1, Ongoing **(Agent in combination with Docetaxel)**	Advanced Solid Tumors Including Castration-Resistant Prostate Cancer	
			NCT01639911, Phase 1, Ongoing **(Agent in combination with Pazopanib)**	Solid Tumors	
			NCT00853307, Phase 2, Completed	Ovarian, Fallopian Tube, or Peritoneal Carcinoma	
			NCT01540682, Phase 1, Completed **(Agent in combination with Cetuximab and radiotherapy)**	Head and Neck Cancer	
			NCT01316692, Phase 2, Ongoing	Unresectable Stage III-IV Melanoma	
			NCT02187991, Phase 2, Ongoing **(Agent in combination with Paclitaxel)**	Metastatic or Locally Recurrent Breast Cancer	
			NCT02259010, Phase 1, Ongoing **(Agent in combination with Itraconazole)**	Advanced Solid Tumors or Relapsed/Refractory Lymphoma	
			NCT02367352, Phase 1, Ongoing **(Agent in combination with Paclitaxel)**	East Asian Patients With Advanced Solid Tumors	
			NCT02319018, Phase 1, Ongoing **(Agent in combination with Leucovorin Calcium, Fluorouracil, and Oxaliplatin)**	Gastrointestinal Tumors	
			NCT02219789, Phase 1, Ongoing **(Agent in combination with Fulvestrant)**	Hormone Receptor Positive, Metastatic or Advanced Breast Cancer	
			NCT02186509, Phase 1, Ongoing **(Agent with radiation)**	Recurrent High-Grade Gliomas	
			NCT01848067, Phase 1&2, Ongoing **(Agent with Abiraterone acetate and Prednisone)**	Hormone-Resistant Prostate Cancer	
			NCT01779843, Phase 1, Ongoing **(Agent with Cytarabine and Idarubicin)**	Acute Myeloid Leukemia	
			NCT02327169, Phase 1, Ongoing **(Agent with MLN2480, MLN0128, and Paclitaxel)**	Advanced Nonhematologic Malignancies	
			NCT00543387, Phase 1, Completed **(Agent in combination with Docetaxel)**	Advanced and/or Refractory Solid Tumors	
MK-5108		AURKA (0.046 nM)	NCT01914510, Phase 2. Ongoing	Ovarian Clear Cell Cancers	
ENMD-2076		AURKA (14 nM)	NCT01719744, Phase 2. Ongoing	Advanced/Metastatic Soft Tissue Sarcoma	
			NCT00904787, Phase 1. Completed	Relapsed or Refractory Hematological Malignancies	
			NCT01639248, Phase 2. Ongoing	Advanced and Metastatic Triple-Negative Breast Cancer	
			NCT00806065, Phase 1. Completed	Multiple Myeloma	
			NCT00658671, Phase 1. Completed	Advanced Cancer	[100]
			NCT02234986, Phase 2, Ongoing	Advanced Fibrolamellar Carcinoma	
			NCT01104675, Phase 2, Completed	Ovarian Cancer	

### ENMD-2076

ENMD-2076 was originally developed by EntreMed, Inc. as a selective AURKA inhibitor with an IC50 of 14 nM in biochemical assays [96]. However, ENMD-2076 was found to inhibit several other oncogenic kinases at different IC50 including FLT3 (1.86 nM), SRC (20.2 nM), VEGFR3 (15.9 nM), VEGFR2 (58.2 nM), PDGFRα (56.4 nM), and FGFR1 (92.7 nM) [96]. This drug was shown to induce G2/M cell cycle arrest and apoptosis, consistent with AURKA inhibition responses. ENMD-2076 was found to be selectively toxic to multiple myeloma (MM) cells but minimally toxic to hematopoietic progenitor cells [97]. Interestingly, exposure as short as 6 hours induces apoptosis and reduces AURKA autophosphorylation at T288; however, this was achieved at doses that also inhibited AURKB and several other kinases [97]. *In vitro* studies in multiple myeloma showed that treatment with ENMD-2076 inhibited AKT phosphorylation, and thus, altered the activity of some of its targets, including GSK3β, survivin, and XIAP [97]. Additionally, ENMD-2076 was shown to down-regulate phosphorylation of STAT3 at Ser-727 [97]. In mouse xenograft models of multiple myeloma, ENMD-2076 showed good tolerability at doses up to 200 mg/kg, and immunohistochemistry analysis revealed reduced Ki67 and increased cleaved caspase-3 expression levels [97]. In a patient-derived xenograft model of CRC, ENMD-2076 inhibited tumor growth, which led to regression; this was associated with significant reduction in 18-FDG uptake at day 3 and 21 of treatment [98]. In breast cancer, ENMD-2076 was most effective against triple-negative tumors with increased mutant p53 expression [99]. In addition, cells lacking estrogen receptor expression and/or HER2 overexpression were the most sensitive cells to the drug [99]. A completed phase 1 clinical trial indicated that this drug is tolerable at 160 mg/m^2^ daily administered orally in patients with advanced solid tumors [100]. ENMD-2076 is currently being evaluated in phase 1 and 2 clinical trials, specifically in breast and ovarian cancers (clinicaltrials.gov, Table 2).

### MK-5108

MK-5018 (VX689) is a specific AURKA inhibitor (IC50: 0.046 nM) developed by Vertex Pharmaceuticals [101]. MK-5018 also inhibits AURKB and AURKC, although at higher doses (200 folds higher than AURKA) with various IC50 values among different cancer cell lines [101]. Tumor xenografts treated with MK-5018 showed significant tumor inhibition at doses of 15 or 30 mg/kg twice daily for 12 days, indicating the high tolerability of the drug [101]. In addition to a typical AURKA inhibition response (polyploidy and cell cycle arrest), inhibition of NF-κB activity and reduction of cytokine production were observed after treatment of ovarian cancer stem cells with MK-5108 [102]. Further, MK-5108 was investigated in combination with other therapeutic agents. For instance, MK-5108, in combination with histone deacetylase inhibitor, vorinostat, induced lymphoma cell death along with reduction of c-Myc, hTERT, and micro RNA levels *in vitro* [103]. In a colon cancer xenograft mouse model, MK-5108 was tested in combination with Docetaxel and showed additive anti-tumor activity as compared to animals that received Docetaxel alone [101]. A completed phase 1 clinical trial of MK-5108 as a single agent or in combination with Docetaxel to test its safety, side effects, and tolerability showed moderate toxicities with some clinical activity as a single agent in patients with advanced and/or refractory solid tumors [104] (clinicaltrials.gov, Table 2).

## Conclusions

Since their discovery, aurora kinases have been under constant investigation to uncover their roles in carcinogenesis. Several studies have indicated that AURKA is overexpressed and/or amplified in different malignances relative to normal cells. A large body of literature has shown that overexpression of AURKA mediates several pro-tumorigenic functions in addition to mitosis; thereby suggesting AURKA as a potential therapeutic target.

Several studies [19,48,49,52,55] identified multiple downstream targets of AURKA involved in critical cellular functions. These studies demonstrated that AURKA not only plays a role in regulating cell cycle and mitosis, but also interacts with a number of key oncogenic signaling networks, suggesting that AURKA is located at a signaling hub in cancer cells. Notably, many of these molecular regulations were established in GI cancers. These findings could be applicable to other types of tumors. However, rigorous investigations are required before a conclusion can be drawn. AURKA activates multiple oncogenic signaling pathways while suppressing critical tumor suppressor functions of p53 [48,89] and p73 [52,56] in cancer cells. AURKA regulates several fundamental properties of carcinogenesis such as cell survival, growth, invasion, inflammation, and angiogenesis. Furthermore, AURKA was shown to regulate pluripotency of embryonic stem cells [13,14], which may suggest that AURKA plays a role in regulating stemness features of cancer cells.

Although our understanding of the role of aurora kinases has improved significantly over the past few years, and several pan or specific Aurora inhibitors were developed with variable specificities, only a few have moved forward in clinical trials. One of the most widely studied inhibitors is MLN8237 (alisertib), which has been investigated in preclinical studies using *in vitro* and tumor xenograft mouse models with promising anti-tumor effects, alone and in combination with Cisplatin [57] or Docetaxel [53]. MLN8237 as single agent or in combination with chemotherapeutics has been evaluated in clinical trials (Table 2). In spite of the extensive preclinical studies on AURKA, few clinical trials were undertaken to evaluate the potentiality of AURKA as a target in GI cancers. The most advanced clinical trial (phase 3) is testing MLN8237 in hematologic malignancies (clinical trial# NCT01482962). In solid tumors, phase 2 clinical trials are currently testing MLN8237 alone or in combination with other chemotherapeutics or biologics (Paclitaxel, Erlotinib, Rituximab, Vincristine among others, Table 2). ENMD-2076 is another specific AURKA inhibitor, currently in phase 2 for ovarian and breast cancers (NCT01914510, NCT01639248, NCT01104675) that has not been tested in GI cancers. Given the experience from other targeted therapy approaches, it is unlikely that AURKA inhibition alone would be sufficient to achieve the desired therapeutic response in solid tumors. Clinical trials utilizing combinations with other anti-cancer drugs may have higher probabilities of success in cancer cell killing by circumventing the expected resistance due to tumor heterogeneity. However, these approaches should be carefully developed as they could be hindered by increased toxicities.

It is also a critical priority to investigate and identify molecular biomarkers of response and resistance to AURKA inhibitors. Biomarker based strategies for patient enrollment into clinical trials hold the future direction for personalized cancer therapy. Based on the available literature, several molecules could be potential biomarkers of response or resistance to AURKA inhibition. These molecules include targets of AURKA such as P53, P73, AKT, BRCA 1&2, STAT3, NF-κB, MDM2, SRC, and MYC [16,17,48,49,52,55,56]. For instance, a study aimed to predict biomarkers of response to the aurora kinase inhibitor, PF-03814735, found that the status of the *MYC* gene family and retinoblastoma pathway members significantly correlated with the efficacy of the inhibitor in small cell lung cancer and colon cancer cell lines [105]. Identification of biomarkers that can reliably predict benefits from AURKA inhibitors should be developed as an integral part of clinical trials in our pursuit for personalized medicine.

In summary, while there is relatively strong biology and several preclinical studies supporting AURKA as an attractive therapeutic target in GI cancers along with the availability of multiple inhibitors, larger and carefully designed clinical trials will be needed to clearly establish the role of AURKA inhibitors in the treatment of GI cancers.

## Abbreviations

GI: Gastrointestinal
AURKA: Aurora kinase A
AURKB: Aurora kinase B
AURKC: Aurora kinase C
TPX2: Targeting protein for Xklp2
BRCA1/2: Breast cancer susceptibility gene
VEGF: Vascular endothelial growth factor
UGI: Upper gastrointestinal
ESCC: Esophageal squamous cell carcinoma
EAC: Esophageal adenocarcinomas
CCND1: Cyclin D1
FGF: Fibroblast growth factor
STAT3: Signal transducer and activator of transcription 3
BCL-xL: B-cell lymphoma-extra large
Bcl2: B-cell lymphoma 2
MCL1: Myeloid Cell Leukemia 1
HDM2: Human double minute 2
PUMA: p53 upregulated modulator of apoptosis
CRC: Colorectal cancer
CHFR: Checkpoint with forkhead and ring finger domains
MAPK: Mitogen-activated protein kinases
ERK: Extracellular signal-regulated kinases
HURP: Hepatoma up-regulated protein
HCC: Human hepatocellular carcinoma
HIF-1: Hypoxia-inducible factor-1
CML: Chronic myeloid leukemia
ALL: Acute lymphoblastic leukemia
AML: Acute myeloid leukemia
MCL: Mantle cell lymphoma
FLT3: FMS-like tyrosine kinase 3
PDGFRα: Alpha-type platelet-derived growth factor receptor
MM: Multiple myeloma
FDG: Fluorodeoxyglucose
HER2: Human epidermal growth factor receptor
hTERT: Human telomerase reverse transcriptase
